# Factors Associated with Short-Term Mortality After Surgical Oncologic Emergencies

**DOI:** 10.1245/s10434-015-4939-8

**Published:** 2015-11-09

**Authors:** Marianne R. F. Bosscher, Esther Bastiaannet, Barbara L. van Leeuwen, Harald J. Hoekstra

**Affiliations:** Department of Surgical Oncology, University Medical Center Groningen, University of Groningen, Groningen, The Netherlands; Department of Surgery, Leiden University Medical Center, Leiden University, Leiden, The Netherlands; Department of Gerontology and Geriatrics, Leiden University Medical Center, Leiden University, Leiden, The Netherlands

## Abstract

**Background:**

The clinical outcome of patients with oncologic emergencies is often poor and mortality is high. It is important to determine which patients may benefit from invasive treatment, and for whom conservative treatment and/or palliative care would be appropriate. In this study, prognostic factors for clinical outcome are identified in order to facilitate the decision-making process for patients with surgical oncologic emergencies.

**Methods:**

This was a prospective registration study for patients over 18 years of age, who were consulted for surgical oncologic emergencies between November 2013 and April 2014. Multiple variables were registered upon emergency consultation, and the follow-up period was 90 days. Multivariate logistic regression analysis was performed to identify factors associated with 30- and 90-day mortality.

**Results:**

During the study period, 207 patients experienced surgical oncologic emergencies—101 (48.8 %) men and 106 (51.2 %) women, with a median age of 64 years (range 19–92). The 30-day mortality was 12.6 % and 90-day mortality was 21.7 %. Factors significantly associated with 30-day mortality were palliative intent of cancer treatment prior to emergency consultation (*p* = 0.006), Eastern Cooperative Oncology Group performance score (ECOG-PS) >0 (*p* for trend: *p* = 0.03), and raised lactate dehydrogenase (LDH) (*p* < 0.001). Additional factors associated with 90-day mortality were low handgrip strength (HGS) (*p* = 0.01) and low albumin (*p* = 0.002).

**Conclusions:**

Defining the intent of prior cancer treatment and the ECOG-PS are of prognostic value when deciding on treatment for patients with surgical oncologic emergencies. Additional measurements of HGS, LDH, and albumin levels can serve as objective parameters to support the clinical assessment of individual prognosis.

An oncologic emergency is an acute condition experienced by a cancer patient that develops directly or indirectly from cancer or cancer treatment.^1^ Surgical procedures may be necessary as a (temporary) remedy.^2^ The clinical outcome of patients with surgical oncologic emergencies is often poor and the short-term mortality is high.^3^–^7^ Surgical treatment can have severe complications and diminish end-of-life quality. It is important to determine which patients may benefit from invasive treatment, and for whom noninvasive treatment or referral to end-of-life care would be appropriate. Unfortunately, patient details are often limited in acute situations.^8^ The heterogeneity of cancer patients and surgical emergencies, as well as the wide range of treatment options, cause difficulties in decision making. Physicians often overestimate the remaining length of life of advanced cancer patients.^9^–^11^

Many studies have tried to identify prognostic factors and create prediction models for survival to assist decision making regarding cancer patients with advanced disease.^10^–^16^ Only a few studies have focused on emergency situations specifically, and even fewer studies have focused on surgical decisions in emergency situations.^17^–^24^ The aim of this study was to establish prognostic factors for the clinical judgment of outcome for patients with surgical oncologic emergencies in order to facilitate the decisional process regarding treatment in the acute setting. In this way, (emergency) physicians would be able to identify patients with short life expectancy, with a minimal amount of information. For this reason, parameters that do not require complex diagnostic procedures were selected for investigation. Thirty-day and ninety-day mortality were chosen as the primary and secondary endpoints.

## Methods

A prospective registration and follow-up was performed for adult cancer patients (age > 18 years) in the University Medical Center Groningen who required surgical consultation for oncologic emergencies between 1 November 2013 and 30 April 2014. The protocol was consistent with the Declaration of Helsinki, and approval for the study was retrieved from the institutional Medical Ethics Committee.

Criteria for inclusion were consultation for surgical oncologic emergencies, which were defined as symptoms related to malignant disease or (previous) cancer treatment for which nonelective surgical consultation and/or admission was required. Patients who were consulted in the emergency room (ER), nonelectively admitted through the (surgical) outpatient clinic, transferred from other hospitals, and who required in-hospital surgical consultation when admitted for nonsurgical specialties were analyzed to meet the inclusion criteria.

Pre-existent patient characteristics were documented as parameters for disease and functional status: sex, age, oncological history, previous cancer treatment, disease status before the emergency consultation, intention of last cancer treatment, body mass index (BMI), the American Society of Anesthesiologists (ASA) classification, and the Eastern Cooperative Oncology Group performance score (ECOG-PS). The ECOG-PS has a stronger association with survival when compared with the Karnofsky Performance Status, and provides better differentiation between ambulatory and bed-ridden patients.^25^ The intent of cancer treatment was regarded as palliative when the patient was documented to have incurable malignant disease.

Following admission (within a maximum of 72 h), parameters as proxies of illness were documented: serum leukocyte count, C-reactive protein (CRP), hemoglobin level, thrombocytes, albumin and lactate dehydrogenase (LDH). As a parameter of muscle strength, the average of three consecutive handgrip strength (HGS) measurements of the right and left hands was documented using the Jamar^®^ Plus+ dynamometer (Sammons Preston, Bolingbrook, IL, USA), which has been found to be more accurate for HGS in advanced cancer patients compared with other dynamometers.^26^ The deviation compared with the normative value for HGS according to age and sex was calculated for each patient, and a combination of both hands was subsequently divided into three categories: low (less than or equal to −4.2 kg deviation), intermediate (between −4.1 and 1.2 kg deviation), and high (≥1.2 kg deviation). When patients had undergone surgical treatment or other types of interventions before the tests could have been performed, the results were not included in the analysis since these could have been influenced by the treatment (in a positive or negative manner).

The final diagnoses of all patients were classified into different categories: obstruction, infection, clinical deterioration, gastrointestinal perforation, bleeding/thrombosis, pathological fractures, and other.^2^ Wound infections were scored according to the Southamptom Wound Assessment Scale.^27^ Intestinal obstruction with clinical evidence of tumor presence was regarded as malignant obstruction, and all other cases of (transient) intestinal obstruction in the absence of signs of disease activity were regarded as benign.

The follow-up period was 90 days after inclusion. At final follow-up, the patients’ charts were analyzed for 30- and 90-day mortality, and all data were processed using IBM SPSS Statistics 22 for statistical analysis (IBM Corporation, Armonk, NY, USA). The four categories of diagnoses with the highest 90-day mortality were selected; one-way analysis of variance (ANOVA) tests, a Kaplan–Meier plot, and log-rank tests were performed to compare means of the different parameters and survival within these four different categories. Multivariate logistic regression analysis was performed to identify factors associated with 30- and 90-day mortality for all patients.

## Results

During the study period, a total of 207 patients were included for analysis—101 (48.8 %) males and 106 (51.2 %) females, with a median age of 64 years (range 19–92). The most prominent type of cancer was colorectal carcinoma (26.1 %). Table 1 provides an overview of the baseline characteristics of the 207 patients. Obstruction was the most frequent surgical oncologic emergency (41.6 %), followed by infections (32.4 %) (Table 2). Of all patients, 40.1 % were surgically treated within 30 days after emergency evaluation; the remaining 59.9 % of patients received conservative, nonsurgical treatment.

**Table 1:** Baseline characteristics of cancer patients who required consultation for surgical oncologic emergencies (*N* = 207)

Characteristic	*N*	%
Patient characteristics		
Age (years)		
≤50	42	20.3
50–64	68	32.8
65–74	67	32.4
75+	30	14.5
Sex		
Male	101	48.8
Female	106	51.2
ECOG performance score		
0	57	27.5
1	85	41.1
2	47	22.7
3	14	6.8
4	4	1.9
ASA classification		
1	22	10.6
2	136	65.7
3	49	23.7
Handgrip strength^a^		
Low (≤−4.2)	31	14.9
Intermediate (−4.1 to 1.2)	32	15.5
High (≥1.2)	32	15.5
BMI		
≤19.9	18	8.7
20.0–24.9	68	32.9
25.0–29.9	50	24.1
≥30.0	27	13.0
Cancer characteristics		
Cancer type		
Colorectal carcinoma	54	26.1
Hepatobiliary	18	8.7
Breast cancer	14	6.8
Soft tissue sarcoma/GIST	14	6.8
Neuroendocrine tumor	13	6.3
Melanoma	11	5.3
Cervix carcinoma	8	3.9
Hematologic malignancy	8	3.9
Esophageal carcinoma	7	3.4
Nonmelanoma skin cancer	6	2.9
Lung carcinoma	4	1.9
Prostate carcinoma	3	1.4
Ovarian carcinoma	3	1.4
Gastric carcinoma	2	1.0
Other	7	3.4
Unknown	14	6.8
No cancer diagnosis	21	10.1
Time since cancer diagnosis		
No cancer diagnosis before consultation	21	10.1
<30 days	26	12.6
30 days–6 months	56	27.1
6 months–1 year	20	9.7
1–2 years	13	6.3
2–5 years	41	19.8
>5 years	30	14.5
Documented stage of treatment before surgical oncologic emergency consultation		
No cancer	21	10.1
Active disease	132	63.8
Diagnostic stage	33	15.9
Receiving treatment with curative intent	51	24.6
Palliative stage	48	23.2
NED after being treated for cancer in the past	54	26.1
<30 days	19	9.2
30 days–6 months	10	4.8
6 months–1 year	7	3.4
1–2 years	6	2.9
2–5 years	6	2.9
>5 years	9	4.3
Treatment characteristics		
Time since last cancer treatment		
Continuously	24	11.6
<30 days	62	30.0
30 days–6 months	32	15.5
6 months–1 year	9	4.3
1–2 years	15	7.2
2–5 years	5	2.4
>5 years	12	5.8
No cancer treatment	48	23.2
Intention of treatment prior to emergency consultation		
No cancer	21	10.1
Diagnostic	32	15.5
Curative	49	23.7
Follow-up	57	27.5
Palliative	48	23.2

*ECOG* Eastern Cooperative Oncology Group, *ASA* American Society of Anesthesiologists, *BMI* body mass index, *GIST* gastrointestinal stromal tumor, *NED* no evidence of disease

^a^Based on the deviation of normative values according to age and sex

**Table 2:** Classification of diagnoses of patients with surgical oncologic emergencies, together with the 30- and 90-day mortality of patients within the different classifications

Classification	Diagnosis	*N*	Overall 30-day mortality	Overall 90-day mortality	Surgery <30 days^a^			No surgery <30 days^a^		
					*N* (%)	30-day mortality^b^	90-day mortality^b^	*N* (%)	30-day mortality^b^	90-day mortality^b^
Total		207	26 (12.6)	45 (21.7)	83 (40.1)	9 (10.8)	21 (25.3)	124 (59.9)	17 (13.7)	24 (19.4)
Obstruction		86	10 (11.6)	22 (25.6)	48 (55.8)	5 (10.4)	13 (27.1)	38 (44.2)	5 (13.2)	9 (23.7)
	Malignant	62	9 (14.5)	20 (32.3)	38 (61.3)	4 (10.5)	11 (31.6)	24 (38.8)	5 (20.8)	9 (37.5)
	Malignant colorectal obstruction	22	3 (13.6)	9 (40.9)	16 (72.7)	1 (6.3)	6 (37.5)	6 (27.3)	2 (33.3)	3 (50.0)
	Malignant bile duct obstruction	19	1 (5.3)	4 (21.1)	7 (36.8)	–	1 (14.3)	12 (63.2)	1 (8.3)	3 (25.0)
	Malignant small bowel obstruction	18	4 (22.2)	5 (27.8)	14 (77.8)	3 (21.4)	4 (28.6)	4 (22.2)	1 (25.0)	1 (25.0)
	Malignant airway obstruction	2	–	1 (50.0)	1 (50.0)	–	–	1 (50.0)	–	1 (100)
	Malignant gastroesophageal obstruction	1	1 (100)	1 (100)	–	–	–	1 (100)	1 (100)	1 (100)
	Benign	24	1 (4.2)	2 (8.3)	10 (41.7)	1 (10.0)	2 (20.0)	14 (58.3)	–	–
	Benign colorectal obstruction	8	–	–	1 (12.5)	–	–	7 (87.5)	–	–
	Benign small bowel obstruction	7	1 (14.3)	1 (14.3)	5 (71.4)	1 (20.0)	1 (20.0)	2 (28.6)	–	–
	Radiation enteritis	4	–	1 (25.0)	4 (100)	–	1 (25.0)	–	–	–
	Benign biliary obstruction	3	–	–	–	–	–	3 (100)	–	–
	Benign gastroesophageal obstruction	1	–	–	–	–	–	1 (100)	–	–
	Benign urinary obstruction	1	–	–	–	–	–	1 (100)	–	–
Infection		67	5 (7.5)	9 (13.4)	14 (20.9)	1 (7.1)	4 (28.6)	53 (79.1)	4 (7.6)	5 (9.4)
	Postoperative wound infection, 1 or 2^c^	6	–	1 (16.7)	–	–	–	6 (100)	–	1 (16.7)
	Postoperative wound infection, 3 or 4^c^	17	–	–	2 (11.8)	–	–	15 (88.2)	–	–
	Postoperative would infection, 5^c^	2	2 (100)	2 (100)	1 (50.0)	1 (100)	1 (100)	1 (50.0)	1 (100)	1 (100)
	Infection/neutropenic enterocolitis during chemotherapy	11	2 (18.2)	3 (27.3)	4 (36.4)	–	1 (25.0)	7 (63.6)	2 (28.6)	2 (28.6)
	Fistula formation after surgery	7	–	–	2 (28.6)	–	–	5 (71.4)	–	–
	Intra-abdominal infection after surgery	7	–	–	1 (14.3)	–	–	6 (85.7)	–	–
	Infectious tumor mass	5	1 (20.0)	3 (60.0)	3 (60.0)	–	2 (66.7)	2 (40.0)	1 (50.0)	1 (50.0)
	Wound healing disturbance after radiation therapy and surgery, 1 or 2^c^	3	–	–	1 (33.3)	–		2 (66.7)	–	–
	Wound healing disturbance after radiation therapy and surgery, 3^c^	1	–	–	–	–	–	1 (100)	–	–
	Chronic presacral abscess formation after surgery and radiation therapy	3	–	–	–	–	–	3 (100)	–	–
	Postoperative gastroenteritis	3	–	–	–	–	–	3 (100)	–	–
	Lymphedema/erysipelas	2	–	–	–	–	–	2 (100)	–	–
Clinical deterioration		19	8 (42.1)	10 (52.6)	4 (21.1)	1 (25.0)	1 (25.0)	15 (79.0)	7 (46.7)	9 (0.6)
	Clinical deterioration due to progressive metastatic disease	9	4 (44.4)	5 (55.6)	1 (11.1)	–	–	8 (88.9)	4 (50.0)	5 (62.5)
	Clinical deterioration and pain due to progressive tumor mass	8	3 (37.5)	4 (50.0)	3 (37.5)	1 (33.3)	1 (33.3)	5 (62.5)	2 (40.0)	3 (60.0)
	Clinical deterioration being NED	2	1 (50.0)	1 (50.0)	–	–	–	2 (100)	1 (50.0)	1 (50.0)
Gastrointestinal perforation		12	3 (25.0)	3 (25.0)	9 (75.0)	2 (22.2)	2 (22.2)	3 (25.0)	1 (33.3)	1 (33.3)
	Perforation with presence of tumor mass	7	3 (42.9)	3 (42.9)	6 (85.7)	2 (33.3)	2 (33.3)	1 (14.3)	1 (100)	1 (100)
	Anastomotic leak	5	–	–	3 (60.0)	–	–	2 (40.0)	–	–
Bleeding/thrombosis		12	–	–	4 (33.3)	–	–	8 (66.7)	–	–
	Tumor bleeding	8	–	–	2 (25.0)	–	–	6 (75.0)	–	–
	Paraneoplastic arterial/venous thrombosis	3	–	–	1 (33.3)	–	–	2 (66.7)	–	–
	Postoperative bleeding	1	–	–	1 (100)	–	–	–	–	–
Pathological fracture		5	–	1 (20.0)	3 (60.0)	–	1 (33.3)	2 (40.0)	–	–
	Fracture due to bone metastases	5	–	1 (20.0)	3 (60.0)	–	1 (20.0)	2 (40.0)	–	–
Other		6	–	–	1 (16.7)	–	–	5 (83.3)	–	–
	Lymphadenopathy/malignant swelling	3	–	–	1 (33.3)	–	–	2 (66.7)	–	–
	Chylous leakage postoperative	2	–	–	–	–	–	2 (100)	–	–
	Incidental diagnosis on imaging studies	1	–	–	–	–	–	1 (100)	–	–

*NED* no evidence of disease

^a^ Number and percentage of patients who underwent surgery within 30 days after consultation for a surgical oncologic emergency, or no surgery within the first 30 days

^b^ Percentages within brackets are the percentages within the categories of ‘Surgery’ and ‘No surgery’

^c^ According to the Southampton Wound Assessment Scale

The 30-day mortality for all patients was 12.6 %, and was highest for patients who presented with clinical deterioration (42.1 %), followed by patients who presented with gastrointestinal leak (25.0 %) (Table 2). The 90-day mortality for all patients was 21.7 %, and was highest for patients with clinical deterioration (52.6 %), followed by patients who presented with obstruction (25.6 %). Overall, the 30-day mortality was higher for patients who underwent nonsurgical treatment (13.7 %) compared with patients who underwent surgery within 30 days after consultation for surgical oncologic emergencies (10.8 %). In contrast, the overall 90-day mortality was higher for patients who underwent surgery (25.3 %) compared with patients who did not undergo surgical treatment (19.4 %).

**Fig. 1:**
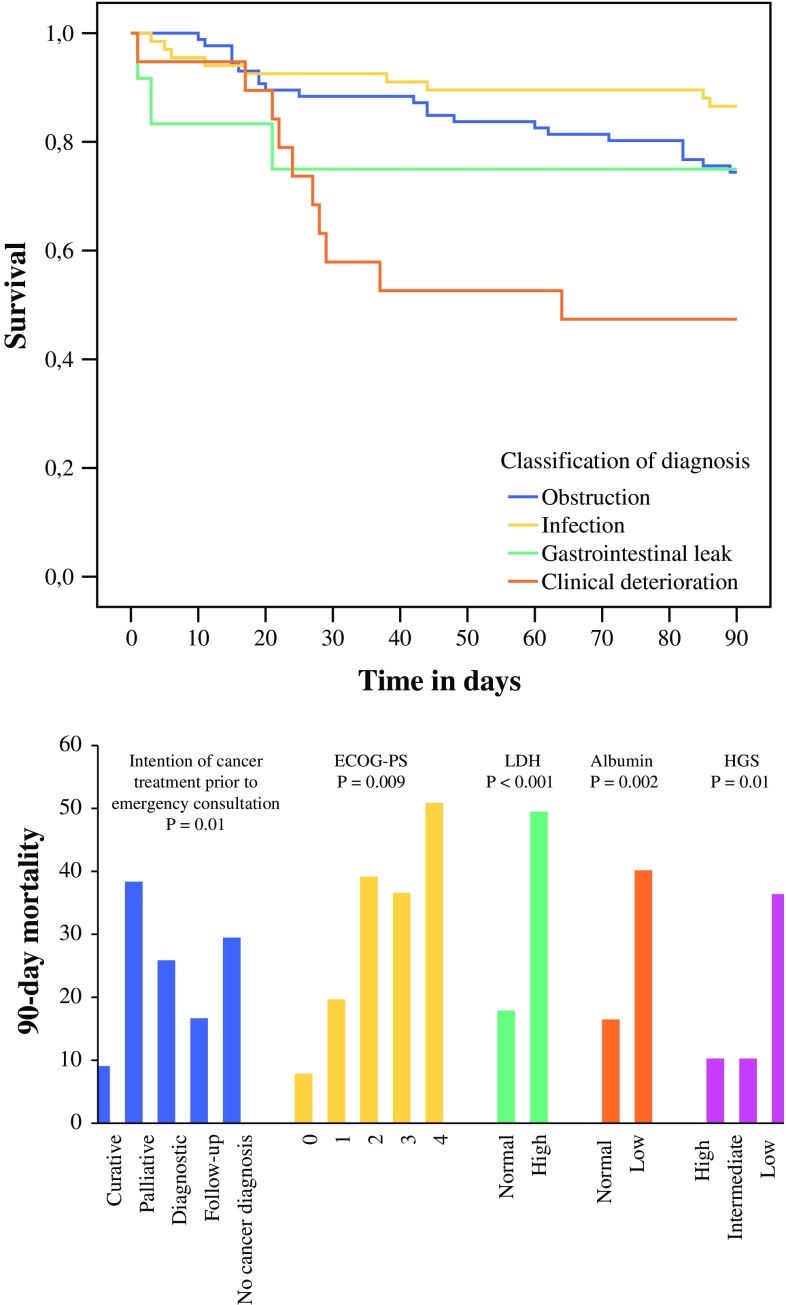
Kaplan–Meier plot for survival of patients with surgical oncologic emergencies, according to the classification of diagnosis, for the classifications with the largest 90-day mortality

The distribution of mortality was statistically different between the different classifications of diagnoses (*p* = 0.002). Of all patients who died after presenting with obstruction, 54.6 % died between 30 and 90 days (Fig. 1).

Table 3 provides an overview of the mean values of the different parameters that were assessed upon inclusion for the four patient groups with the highest 90-day mortality. The mean ECOG-PS (*p* < 0.001), CRP (*p* < 0.001), and LDH levels (*p* = 0.031) were significantly different between the classifications of diagnoses. Other parameters showed no significant difference. The different parameters have been stratified by classification of diagnosis, and high LDH was significantly associated with 90-day mortality independent of age or sex (*p* = 0.01) of the patients who presented with obstruction (*p* = 0.02) or infection (*p* = 0.003). Other parameters showed no association for specific diagnoses.

**Table 3:** Mean values of the different parameters according to the classification of diagnosis, for the classifications with the largest 90-day mortality

Classification	Obstruction	Infection	Clinical deterioration	Gastrointestinal leak	*p* value^a^
*N* (%)	86 (41.6)	67 (32.4)	19 (9.2)	12 (5.8)	
ECOG-PS [Mean (range)]	1.21 (0–4)	0.81 (0–4)	2.16 (1–4)	1.17 (0–2)	0.000
ASA classification [mean (range)]	2.20 (1–3)	2.01 (1–3)	2.16 (1–3)	2.40 (2–3)	0.068
Distress thermometer [mean (range)]	6.01 (0.5–10)	5.56 (1–10)	6.55 (0–10)	6.1 (2–10)	0.784
CRP [mean (range)]	60 (3–420)	120 (0–432)	47 (0–308)	160 (3–385)	0.000
Leukocytes [mean (range)]	11.4 (3.9–38.8)	11.7 (0.1–61.3)	11.5 (5.0–19.8)	13.4 (7.4–21.9)	0.816
Hemoglobin [mean (range)]	7.6 (4.7–11.4)	7.1 (3.2–10.2)	7.7 (5.7–10.0)	7.4 (5.3–9.6)	0.152
Thrombocytes [mean (range)]	349 (133–961)	306 (14–895)	370 (166–559)	333 (146–519)	0.507
Creatinine [mean (range)]	86 (29–412)	94 (35–525)	81 (24–182)	87 (36–305)	0.849
LDH [mean (range)]	210 (0–1236)	246 (0–2180)	405 (142–1509)	214 (122–303)	0.031
Albumin [mean (range)]	36 (19–49)	33 (16–49)	36 (21–45)	31 (17–45)	0.064
HGS L^b^ [mean (range)]	−0.3 (−22.6–18.6)	−4.5 (−18.3–3.2)	−2.9 (−14.7–9.5)	−1.8 (−3.7–0.2)	0.308
HGS R^b^ [mean (range)]	−3.4 (−28.0–19.0)	−6.9 (−27.4–12.3)	−7.7 (−20.1–4.1)	−6.8 (−7.6–−5.6)	0.365
BMI [mean (range)]	25.1 (15.4–39.0)	26.4 (18.4–41.6)	24.6 (17.5–32.4)	27.6 (21.5–45.9)	0.329

*ECOG*-*PS* Eastern Cooperative Oncology Group performance score, *ASA* American Society of Anesthesiologists, *CRP* C-reactiveprotein, *LDH* lactate dehydrogenase, *HGS L* hand-grip strength, left-hand, *HGS R* hand-grip strength, right-hand, *BMI* body mass index, *ANOVA* analysis of variance

^a^ One-way ANOVA

^b^ Based on the deviation of the normative according to age and sex

Table 4 shows the results of the univariate and multivariate analysis of the different variables for 30- and 90-day mortality. Factors significantly associated with 30-day mortality were palliative intent of treatment prior to emergency consultation (*p* = 0.008), an ECOG-PS > 0 (*p* for trend: *p* = 0.03), and raised LDH (*p* < 0.001). The remaining parameters showed no association with 30-day mortality. Factors significantly associated with 90-day mortality were palliative treatment prior to emergency consultation (*p* = 0.01), ECOG-PS > 0 (*p* = 0.003), low HGS (less than or equal to −4.2 kg deviation of the normative value; *p* = 0.01), raised LDH (*p* < 0.001), and low albumin levels (*p* = 0.002). All these factors remained significant after adjustment for age and sex.

**Table 4:** Factors associated with 30- and 90-day mortality after surgical oncologic emergencies

Factor	30-day mortality	OR (95 % CI)	*p* value	Multivariable OR (95 % CI)^a^	*p* value	90-day mortality	OR (95 % CI)	*p* value	Multivariable OR (95 % CI)^a^	*p* value
Patient characteristics										
Age^b^										
≤50	9.5	1.0 (ref)	0.5			16.7	1.0 (ref)	0.2		
50–64	8.8	0.9 (0.2–3.5)				20.6	1.3 (0.5–3.5)			
65–74	16.4	1.9 (0.6–6.3)				19.4	1.2 (0.4–3.3)			
75+	16.7	1.9 (0.5–7.8)				36.7	2.9 (1.0–8.7)			
Sex										
Male	13.9	1.0 (ref)	0.6			23.8	1.0 (ref)	0.5		
Female	11.3	0.8 (0.3–1.8)				19.8	0.8 (0.4–1.5)			
ECOG-PS										
0	1.8	1.0 (ref)	0.03	1.0 (ref)	0.03	7.0	1.0 (ref)	0.003	1.0 (ref)	0.009
1	11.8	7.5 (0.9–60.0)		7.0 (0.8–59.1)		18.8	3.1 (1.0–9.7)		3.2 (1.0–10.7)	
2	21.3	15.1 (1.9–123)		14.3 (1.6–125)		38.3	8.2 (2.5–26.6)		7.9 (2.3–27.8)	
3	21.4	15.3 (1.5–160)		17.3 (1.5–203)		35.7	7.4 (1.6–32.7)		6.6 (1.3–32.2)	
4	50.0	56.0 (3.4–906)		59.0 (3.5–985)		50.0	13.2 (1.4–120)		13.7 (1.5–126)	
ASA classification										
1	4.6	1.0 (ref)	0.4			4.6	1.0 (ref)	0.1		
2	12.5	3.0 (0.4–23.8)				22.1	5.9 (0.8–46.0)			
3	16.3	4.1 (0.5–34.9)				28.6	8.4 (1.0–68.5)			
Handgrip strength										
High	6.3	1.0 (ref)	0.4			9.4	1.0 (ref)	0.01	1.0 (ref)	0.01
Intermediate	3.1	0.5 (0.04–5.6)				9.4	1.0 (0.2–5.4)		1.2 (0.2–7.0)	
Low	12.9	2.2 (0.4–13.1)				35.5	5.3 (1.3–21.5)		6.9 (1.6–29.4)	
BMI										
Normal (20.0–24.9)	10.3	1.0 (ref)	0.4	1.0 (ref)	0.4	23.5	1.0 (ref)	0.4		
Low (≤ 19.9)	5.6	0.5 (0.1–4.4)		0.6 (0.1–5.2)		16.7	0.7 (0.2–2.5)			
Overweight (25–29.9)	6.0	0.6 (0.1–2.3)		0.5 (0.1–2.1)		12.0	0.4 (0.2–1.2)			
Obese (≥ 30.0)	18.5	2.0 (0.6–6.9)		1.9 (0.5–6.7)		22.2	0.9 (0.3–2.7)			
Treatment characteristics										
Intention of treatment prior to emergency consultation										
Curative	4.1	1.0 (ref)	0.008	1.0 (ref)	0.006	8.2	1.0 (ref)	0.01	1.0 (ref)	0.01
Palliative	29.2	9.7 (2.1–45.4)		9.8 (2.0–47.2)		37.5	6.8 (2.1–21.9)		6.8 (2.0–22.6)	
Diagnostic	9.4	2.4 (0.4–15.4)		2.3 (0.4–15.1)		25.0	3.8 (1.0–13.7)		3.6 (1.0–13.5)	
Follow-up	8.8	2.3 (0.4–12.2)		1.9 (0.3–11.0)		15.8	2.1 (0.6–7.3)		1.9 (0.5–6.8)	
No cancer	9.5	2.5 (0.3–18.9)		2.0 (0.2–15.7)		28.6	4.5 (1.1–18.1)		3.4 (0.8–14.3)	
Leukocytes										
Normal (4–10)	12.5	1.0 (ref)	0.9			21.3	1.0 (ref)	0.9		
Low	12.5	1.0 (0.1–9.0)				25.0	1.2 (0.2–6.7)			
High	13.5	1.1 (0.5–2.6)				23.1	1.1 (0.6–2.2)			
Hemoglobin										
Normal	12.5	1.0 (ref)	0.9			18.7	1.0 (ref)	0.4		
Low	13.3	1.1 (0.4–2.6)				24.2	1.4 (0.6–2.9)			
CRP										
Normal	12.5	1.0 (ref)				12.5	1.0 (ref)	0.1		
High	13.8	1.1 (0.4–3.5)	0.8			24.5	2.3 (0.7–6.9)			
Thrombocytes										
Normal (<350)	11.7	1.0 (ref)	0.3			20.7	1.0 (ref)	0.3		
High	17.4	1.6 (0.7–3.7)				27.5	1.5 (0.7–2.9)			
Creatinine										
Normal	13.0	1.0 (ref)	0.7			22.7	1.0 (ref)	0.8		
High	15.2	1.2 (0.4–3.5)				24.2	1.1 (0.5–2.6)			
LDH										
Normal	8.2	1.0 (ref)	<0.001	1.0 (ref)	<0.001	17.0	1.0 (ref)	<0.001	1.0 (ref)	<0.001
High	35.1	6.1 (2.5–14.9)		6.2 (2.5–15.3)		48.6	4.6 (2.1–10.0)		4.8 (2.2–10.6)	
Albumin										
Normal	10.4	1.0 (ref)	0.08	1.0 (ref)	0.06	15.6	1.0 (ref)	0.002	1.0 (ref)	0.002
Low	21.3	2.3 (0.9–6.1)		2.6 (1.0–6.8)		39.3	3.5 (1.6–7.8)		3.9 (1.7–9.0)	

*OR* odds ratio, *CI* confidence interval, *ECOG*-*PS* Eastern Cooperative Oncology Group performance score, *ASA* American Society of Anesthesiologists, *HGS* handgrip strength, *BMI* body mass index, *CRP* C-reactive protein, *LDH* lactate dehydrogenase

^a^ Age and sex adjusted OR

^b^ Age continue: OR 1.03 (0.99–1.05; *p* = 0.065)

## Discussion

In this study, the 30- and 90-day mortality for all patients who required consultation for surgical oncologic emergencies was 12.6 and 21.7 %, respectively. Factors that were significantly associated with 30-day mortality were pre-existent palliative intent of treatment, an ECOG-PS > 0, and raised LDH. Additionally, low albumin levels and low HGS were associated with 90-day mortality. These factors can all be seen as parameters of decreased functional status (i.e., performance), malnutrition, and/or advanced cancer, which are generally associated with decreased quality of life and survival.^28^–^31^

Advanced cancer (receiving treatment with palliative intent) and raised ECOG-PS (>0) were significantly associated with 30-day mortality. Although not specifically in an acute setting, the ECOG-PS and other functional status classification systems have already shown to be correlated with stage of disease and to be a strong predictor of survival for patients with advanced cancer.^10^,^12^,^14^,^22^,^25^,^32^–^34^ In one study, the ECOG-PS was the strongest predictor for mortality for patients with stage IV cancer and malignant bowel obstruction, and the median survival decreased from 222 to 63 days for patients with an ECOG-PS > 1.^22^ These results underscore the importance of defining the cancer stage and functional status of a cancer patient when deciding on treatment, especially in an acute setting.

Since time is often scarce, there is a need for objective parameters that could easily be measured in order to assist in estimating the performance and predicting the clinical outcome of surgical oncologic emergencies. When patients undergo invasive treatment such as surgery, it is essential that the patient is able to recover from this invasive procedure and that the procedure itself does not reduce the patient’s quality of life.

Blood tests and other laboratory tests are often routinely performed, especially for patients who visit the ER. In this study, raised LDH was significantly associated with 30-day mortality and low albumin was associated with 90-day mortality after surgical oncologic emergencies. The prognostic value of raised LDH and low albumin for terminally ill cancer has been confirmed by other studies; however, it has not been widely investigated in an acute setting.^22^,^25^,^30^,^35^–^37^ The remaining blood markers that were analyzed for this study (i.e., leukocytes, hemoglobin, CRP, thrombocytes, and creatinine) were not generally associated with 30- or 90-day mortality after surgical oncologic emergencies.

In general, sepsis (often accompanied with elevated serum CRP levels) after emergency surgery is associated with postoperative mortality.^38^ In this study, the mortality for patients with high CRP levels was lower compared with patients from other groups. Even within the classification of infection, high CRP levels were not associated with 30- or 90-day mortality, possibly due to the fact that most patients within the group of infections had postoperative wound infections, which are often conservatively treated with antibiotics or drainage. Furthermore, the number of patients with severe sepsis was relatively small. Studies on electively treated cancer patients found that CRP is associated with malignant potential and tumor stage, and thus general prognosis.^32^,^39^,^40^ CRP has been found to increase significantly 1–2 weeks prior to death.^41^ In a study evaluating CRP levels in patients with advanced cancer visiting the ER, CRP was considered to be an independent predictor for 14-day mortality, but the specific diagnoses and the effect of antimicrobial treatment were not specified.^23^

In the present study, low HGS (less than or equal to −4.2 kg deviation of the normative value) was associated with 90-day mortality. HGS has been found be a measure of muscle function, cachexia, and malnutrition in several cancer populations, and a better predictor of clinical outcome than measuring the appendicular muscle mass.^28^,^31^,^42^–^48^ HGS has been associated with a significantly lower BMI, hemoglobin, and albumin, and increased ECOG-PS. Malnutrition and low HGS have further been associated with an increased length of hospital stay and mortality. In one study, patients with a decline of HGS to less than a 10th percentile of normative values had statistically shorter survival compared with patients with higher HGS, independent of age, sex, oncological treatment, and cancer type.^28^,^43^ Nevertheless, HGS less than a 10th percentile of the normative value is an extensive decline in strength, and the survival period was relatively long. The current study has found that even a smaller decline in HGS (less than or equal to −4.2 kg deviation of the normative value) was already associated with 90-day mortality.

The results of both previous studies and the current study confirm that HGS can be seen as a measurement of functional status and that low HGS is associated with poor clinical outcome. To our knowledge, this is the first study evaluating the clinical value of HGS specifically for patients who are consulted for surgical oncologic emergencies. Early measurement of HGS in patients with surgical oncologic emergencies can be of value in order to identify patients with advanced cancer and poor functional status who require referral to palliative care. When dynamometers become generally available at the ER and hospital wards, HGS could be an easy measure for patients who require prompt decisions. Unfortunately, HGS could not be measured prior to treatment in every patient. Many patients were admitted and treated outside office hours when research personnel were not available, and some patients were not hospitalized after visiting the ER. For other patients, the measurements were not performed at the time of the first consultation but after 1 or 2 days of admission, and therefore the hand grip strength could have been influenced. Possibly, when more HGS measurements would have been performed over a shorter term after the first consultation, a stronger association with short-term mortality may have been found. Further studies are necessary to validate the prognostic value of HGS in the emergency setting.

Clinical functional status scoring systems and other parameters have already been incorporated in multiple prediction models for survival of terminally ill cancer patients^10^,^25^,^36^,^49^; however, none of these models have been evaluated for patients with (surgical) oncologic emergencies. The results of this study confirm that defining the ECOG-PS and cancer stage, as well as measurements of LDH, albumin, and HGS, are of prognostic value for patients with surgical oncologic emergencies with respect to 30- and 90-day mortality. When deciding on the extent of treatment, the main goal of treatment should be (temporary) solution of the emergency without reducing survival or quality of life. Being able to recognize patients who are at the end of life could prevent unnecessary investigations and expensive treatment, and preserve overall patient satisfaction. The parameters investigated in this study will not predict a specific remaining duration of life, but the combination of prognostic factors found in this study can support the clinical judgment of a physician who is confronted with surgical oncologic emergencies.

Only two other studies have investigated factors associated with mortality in patients with surgical oncologic emergencies.^17^,^24^ Dumont et al. created a preoperative normogram for decision making in surgical oncologic emergencies, which included the ECOG-PS and albumin level (both confirmed by the current study), as well as the Portsmouth Physiological and Operative Severity Score for the enumeration of Mortality and Morbidity (P-POSSUM).^17^ The major drawback of the P-POSSUM is that this score is not designed for the acute setting and requires comprehensive preoperative diagnostic studies. A study by Roses et al. has identified ASA classification >3 and albumin as independent predictors for 30-day mortality.^24^ Active malignant disease, a tumor-related emergency, ASA > 3, and raised creatinine were independent predictors of decreased overall survival. For patients in the current study, the pre-existent ASA classification was assessed and did not include any patient classified higher than 3. The ASA classification was not associated with short-term mortality. The ECOG-PS seems to be a better indicator for clinical outcome in the acute setting.

The cohort of patients in this study represents a very heterogeneous population, and patients experienced a wide range of emergencies with various severities. As this was an observational study, patients were not selected or randomized for invasive or noninvasive treatment according to the prognostic factors. The treatment instituted for each patient was dependent on the decisions of the physicians involved during admission, and was not influenced by this study. Furthermore, because of the amount of missing values and the relatively small numbers of patients in the different subgroups, we were not able to create a solid prediction model. For this reason, only factors that showed significant association with 30- and 90-day mortality were shown. However, we believe that the combination of these parameters can assist in estimating a patient’s prognosis in the acute setting.

## Conclusions

Consultation for surgical oncologic emergencies can be a sign of advanced disease, and outcome is often poor. Being able to recognize patients who are at the end of life would prevent unnecessary investigations, expensive treatment, and preserve patient satisfaction. There is a need for parameters that can easily be measured in order to assist in predicting the clinical outcome. Defining the intention of prior cancer treatment and the ECOG-PS are of prognostic value when deciding on the extent of treatment for patients with surgical oncologic emergencies. Additional measurements of LDH and albumin levels, as well as HGS, can serve as objective prognostic parameters to assist the clinical judgment of individual outcome. This would aid the decision-making process in the acute setting. In this way, patient-tailored treatment can be instituted and overtreatment can be prevented. Further studies will be necessary to validate the prognostic value of these and possible additional parameters in the acute oncology setting.
